# Supplementary material for: A mutational atlas for Parkin proteostasis
Source: Nat Commun. 2024 Feb 20;15:1541. doi: 10.1038/s41467-024-45829-4 (PMC10879094; doi:10.1038/s41467-024-45829-4)
Supplement: Supplementary file 1 — Supplementary Information [file 41467_2024_45829_MOESM1_ESM.pdf]

A mutational atlas for Parkin proteostasis

Supplementary Information

Sup. Fig. 1, GFP-Parkin is functional in HEK293T cells. p.2
Sup. Fig. 2, Correlations between independent repeats. p.3
Sup. Fig. 3, Correlations with the relative abundance of Parkin variants in U2OS cells. p.4
Sup. Fig. 4, Surface exposed beta strands display alternating mutational sensitivity. p.5
Sup. Fig. 5, Zn^{2+} coordinating residues are sensitive to mutations. p.6
Sup. Fig. 6, High abundance variants are positioned in exposed regions. p.7
Sup. Fig. 7, Positioning of low abundance variants in the Parkin structure. p.8
Sup. Fig. 8, Stabilization of Parkin by mutation of the active site. p.9
Sup. Fig. 9, Hyperactive Parkin variants display a reduced abundance. p.10
Sup. Fig. 10, Low stability tiles are buried in the Parkin structure. p.11
Sup. Fig. 11, Effect of mutations in a buried Parkin tile. p.12
Sup. Fig. 12, Predicted degradation signals in low-abundance variants. p.13
Sup. Fig. 13, Side-by-side comparison of the abundance map with the computational maps. p.14
Sup. Fig. 14, Correlation with Parkin melting temperatures. p.16
Sup. Fig. 15, Functional model classes mapped to the Parkin structure. p.17
Sup. Fig. 16, Positioning of the pathogenic variants in the Parkin structure. p.18
Sup. Fig. 17, Correlations with EVE predictions. p.19
Sup. Fig. 18, Distribution of the number of nucleotide substitutions in the library. p.20
Sup. Fig. 19, Flow cytometry gating strategy. p.21

Sup. Table 1, Data for the pathogenic and benign variants included in this study. p.22
Sup. Table 2, Primers used in this study. p.23

References for supplementary information p.24

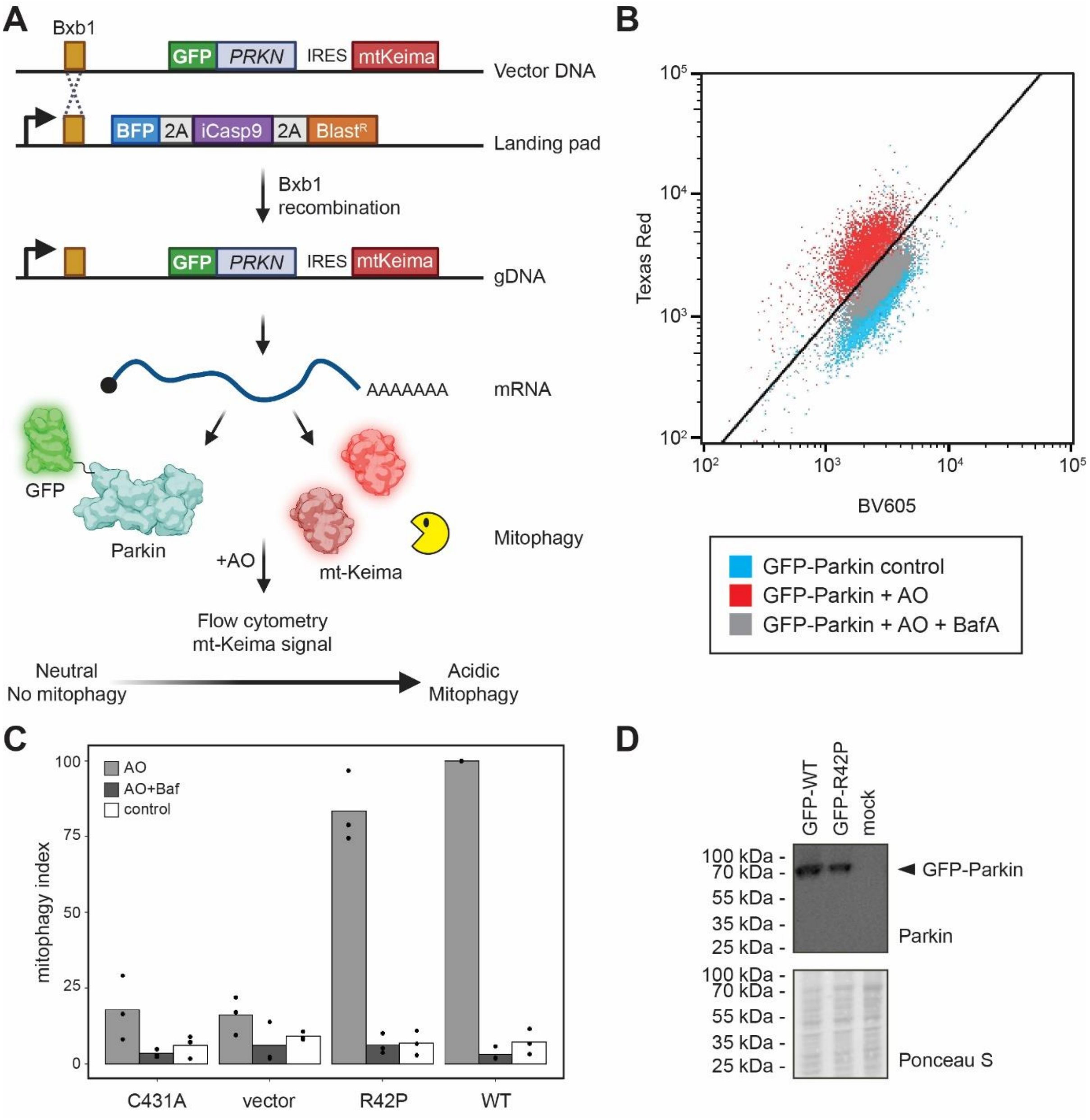

Supplementary Figure 1 –*GFP-Parkin is functional in HEK293T cells.*

(A) Schematic representation of the expression system. GFP-fused *PRKN* followed by an internal ribosomal entry site (IRES) and the mitophagy reporter mt-Keima was introduced into the landing pad in HEK293T cells by Bxb1 specific recombination. After correct integration, GFP-Parkin and mt-Keima are expressed from the same mRNA. Mitophagy is induced by addition of antimycin and oligomycin (AO) and measured by flow cytometry. Figure created with BioRender.com. (B) Representative flow cytometry scatter plots of landing pad cells expressing GFP-Parkin (WT) either untreated (blue, n=7,332 cells), treated with AO to induce mitophagy (red, n=6,723 cells), or treated with both AO and the inhibitor bafilomycin A (BafA) (grey, n=6,215 cells) (Texas Red indicates acidic Keima, BV605 indicates neutral Keima). (C) Quantification of the indicated Parkin variants ability to induce mitophagy based on flow cytometry as shown in panels A and B (n=3). Note that WT and R42P GFP-fused Parkin are active, while the catalytically dead C431A variant is not (similar to empty vector control). (D) Expression of Parkin in the HEK293T landing pad cell line was analyzed by SDS-PAGE and western blotting using antibodies to Parkin. Ponceau S staining was used as a loading control. Note that we were unable to detect any endogenous Parkin in the cells and the R42P variant is thus overexpressed.

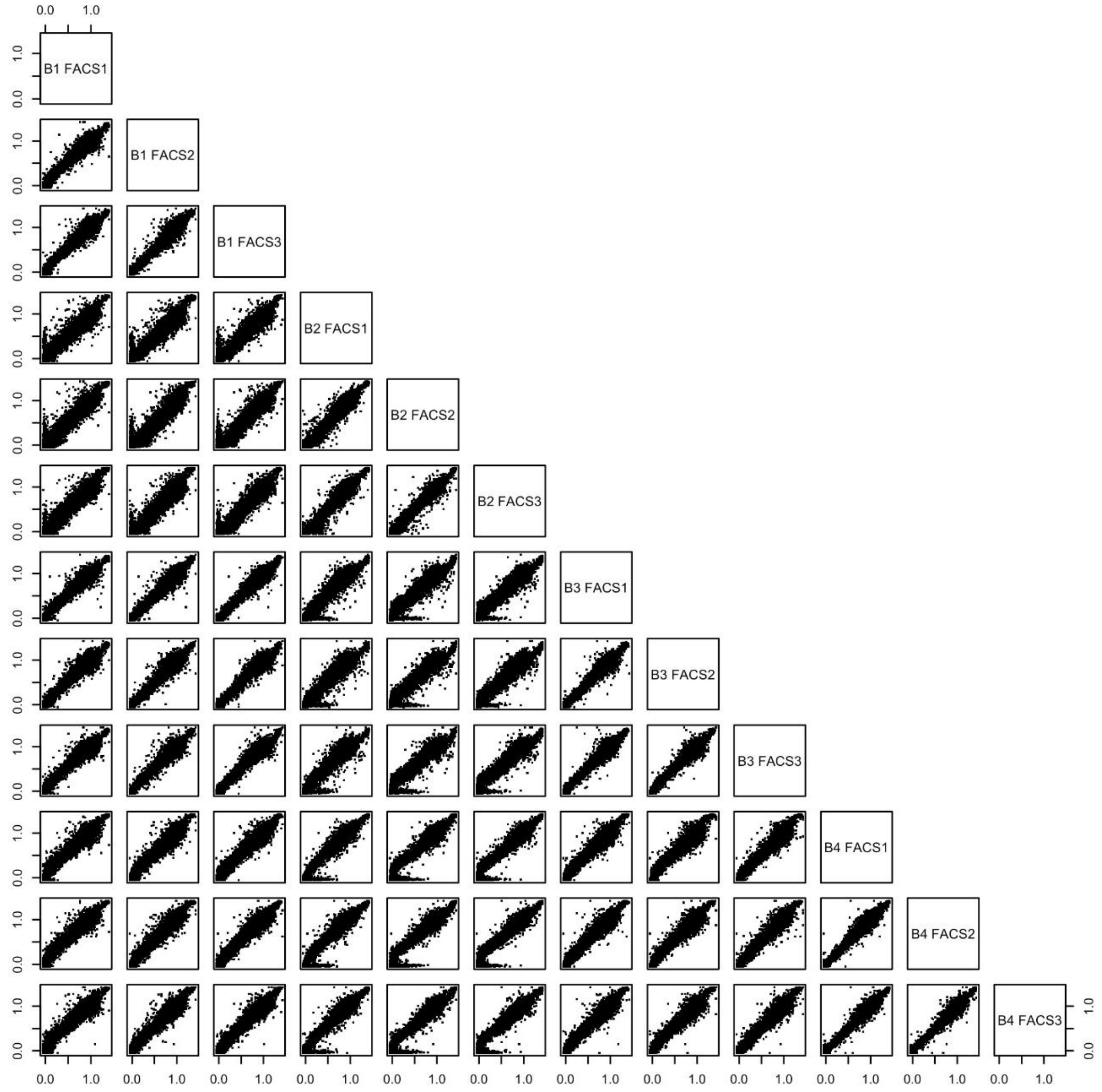

Supplementary Figure 2 – *Correlations between independent repeats.*

Reproduction of single amino acid variant scores between all four biological replicates (B1-B4) each with three FACS replicates. All Pearson correlations are in the range 0.96 to 0.99.

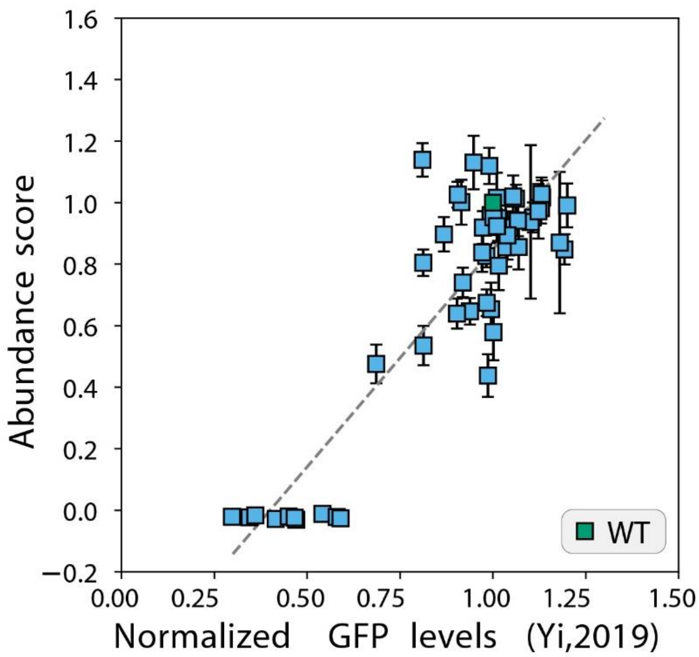

Supplementary Figure 3 – *Correlations with the relative abundance of Parkin variants in U2OS cells.* Correlation of Parkin variant abundance with their relative abundance in U2OS cells based on fluorescence microscopy [1]. The error bars indicate the standard deviation. The Spearman's r is: 0.61.

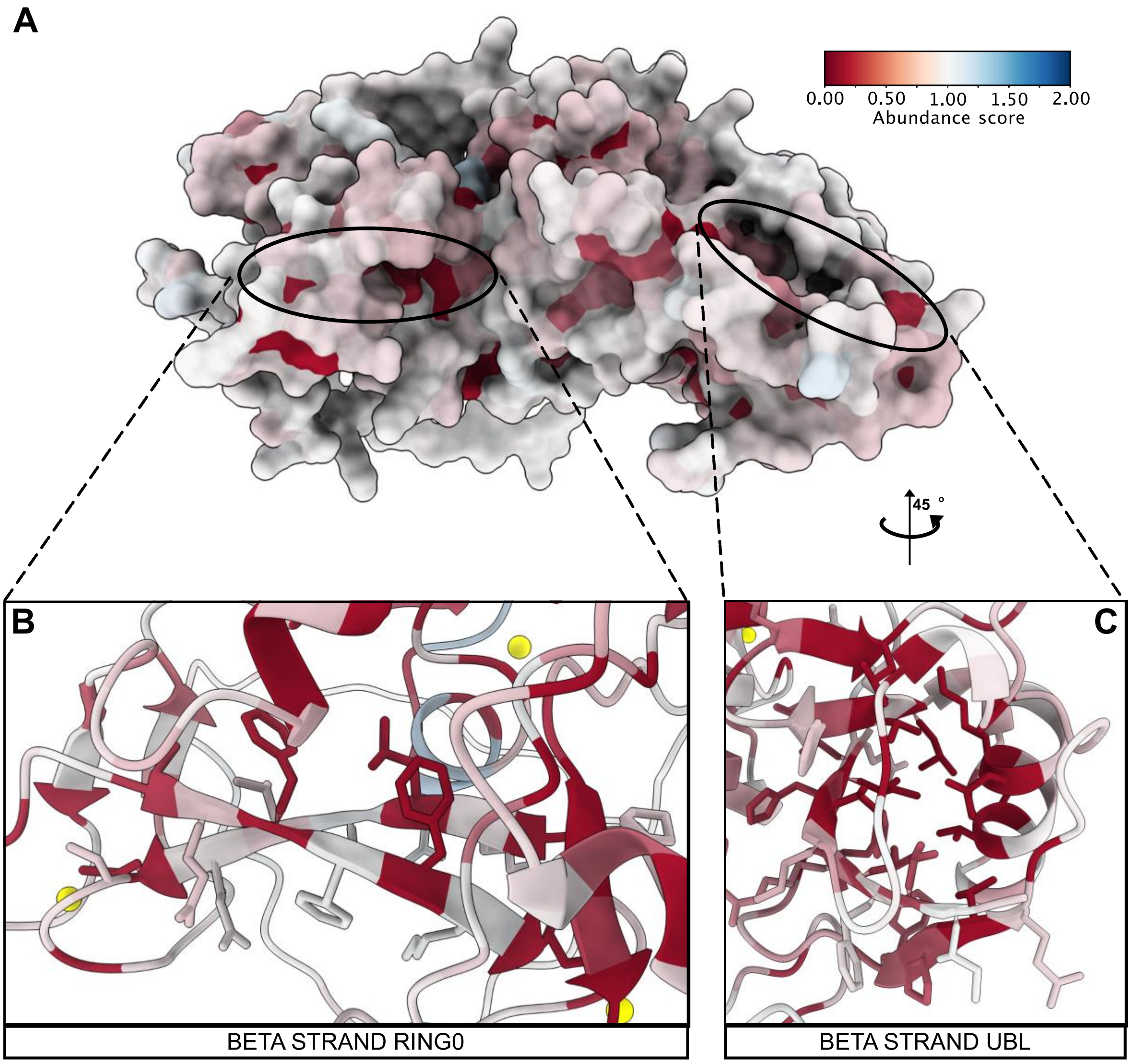

Supplementary Figure 4 – *Surface exposed beta strands display alternating mutational sensitivity.* Examples of the alternating mutational patterning of the β-strands are shown for the RING0 (AB) and UBL (AC) domains. The structure is colored based on the median abundance score per residue.

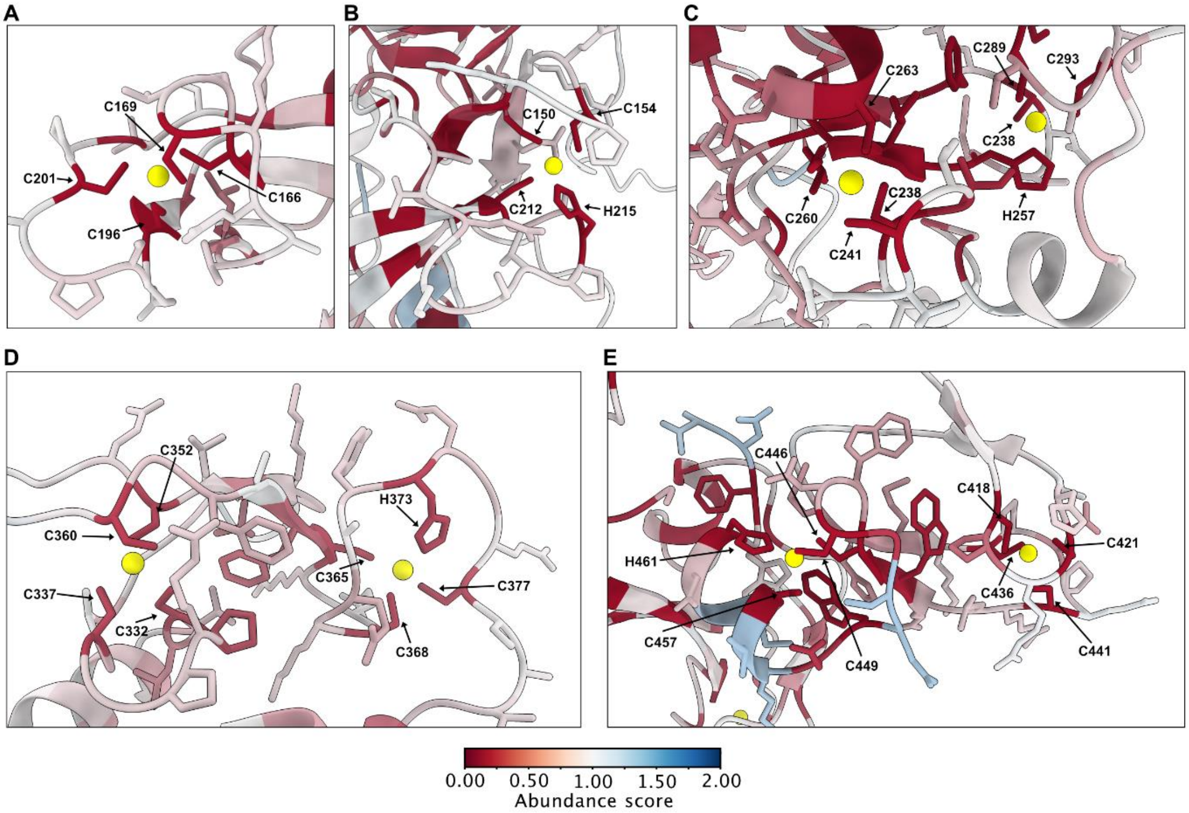

Supplementary Figure 5 – *Zn^{2+} coordinating residues are sensitive to mutations.*
Zoom-in views on the Zn^{2+} binding residues in the Parkin structure in the (AB) RING0 domain, (C) the RING1 domain, (D) IBR domain and (E) RING2 domain. The structure is colored based on the median abundance score per residue.

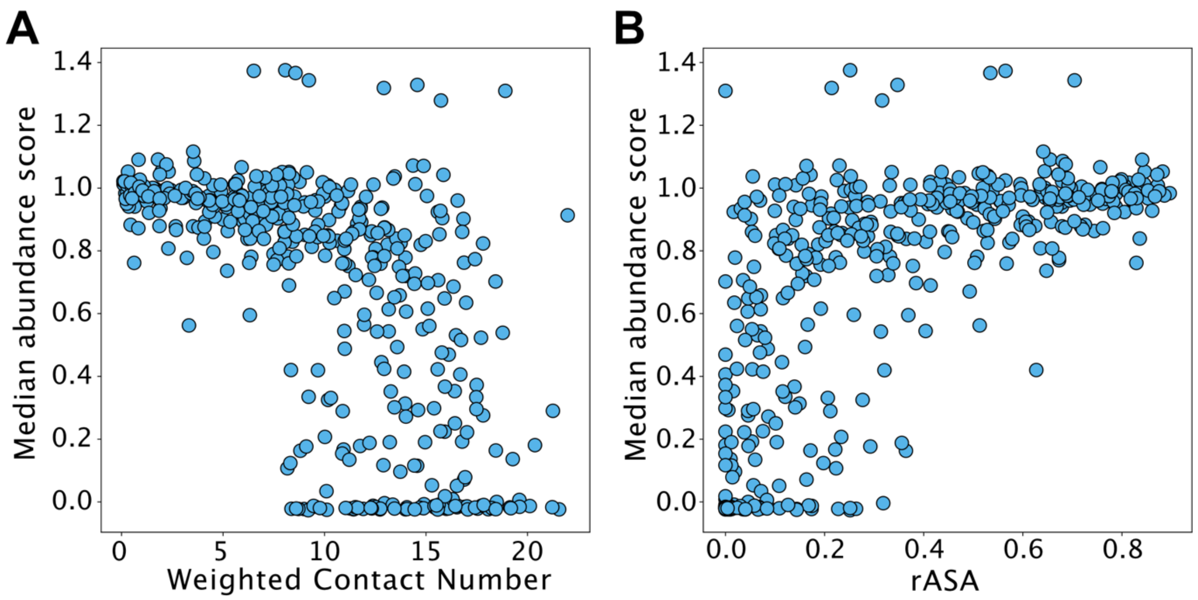

Supplementary Figure 6 - *High abundance variants are positioned in exposed regions.*

Plot of the median abundance score per position against (A) the weighted contact number (WCN) and (B) the relative accessible surface area (rASA). The Spearman's r is: -0.66 in panel (A) and 0.71 for panel (B).

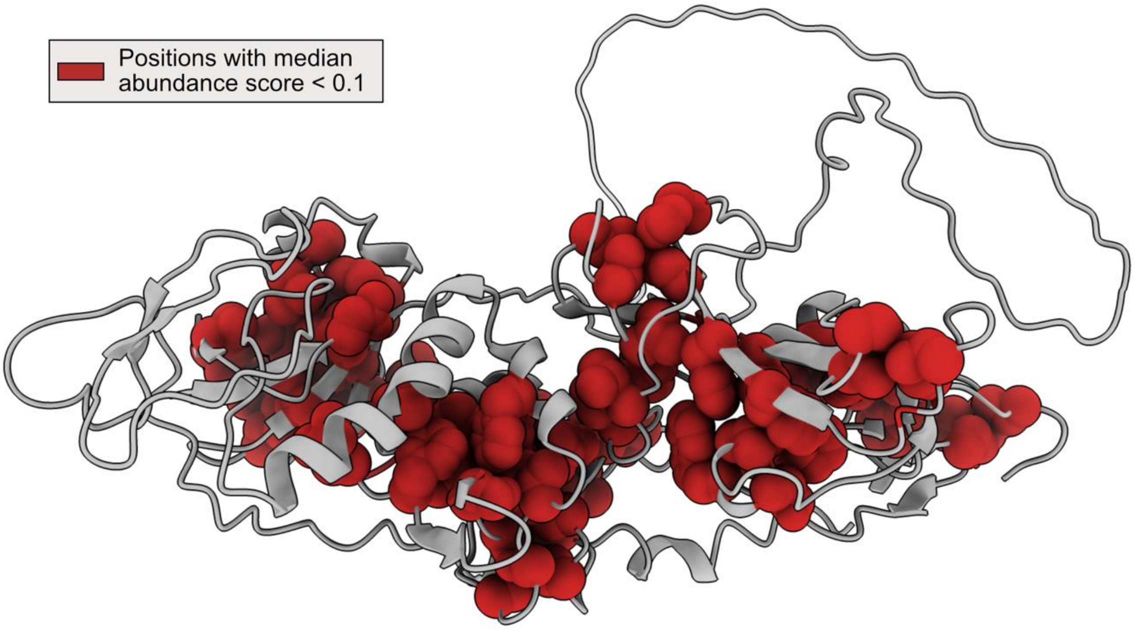

Supplementary Figure 7 – *Positioning of low abundance variants in the Parkin structure.*
Cartoon representation of the Parkin structure with position with median low abundance (<0.1) marked with van der Walls spherical representation in red.

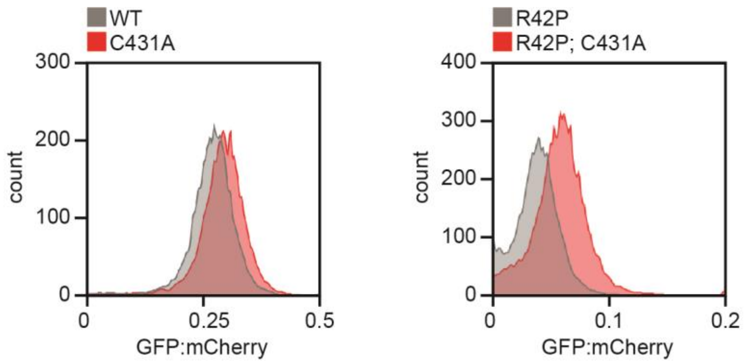

Supplementary Figure 8 – *Stabilization of Parkin by mutation of the active site.*
Flow cytometry profiles displaying wild-type (WT), catalytically dead (C431A), low abundance (R42P), and double mutant (R42P C431A) Parkin variants. Note that the R42P C431A double mutant displays a slightly increased level compared to the R42P single variant. Note that the scale on the x-axis is linear and the stabilization conferred by the C431A mutation is minor.

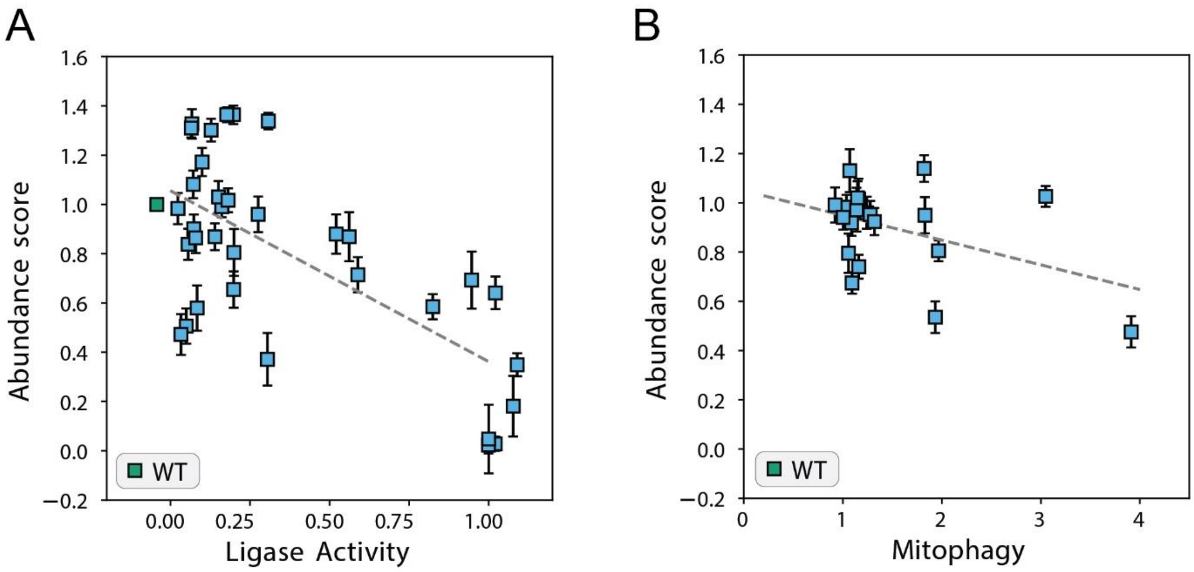

Supplementary Figure 9 – *Hyperactive Parkin variants display a reduced abundance.*
(A) Correlation of Parkin abundance scores with the relative activity of hyperactive Parkin variants evaluated by *in vitro* ubiquitination assays [2]. The Spearman's r is: -0.48. (B) Correlation of Parkin abundance scores with the relative CCCP-induced mitophagy activity of hyperactive Parkin variants normalized to their abundance [1]. The Spearman's r is: -0.20. Error bars indicate the standard deviation.

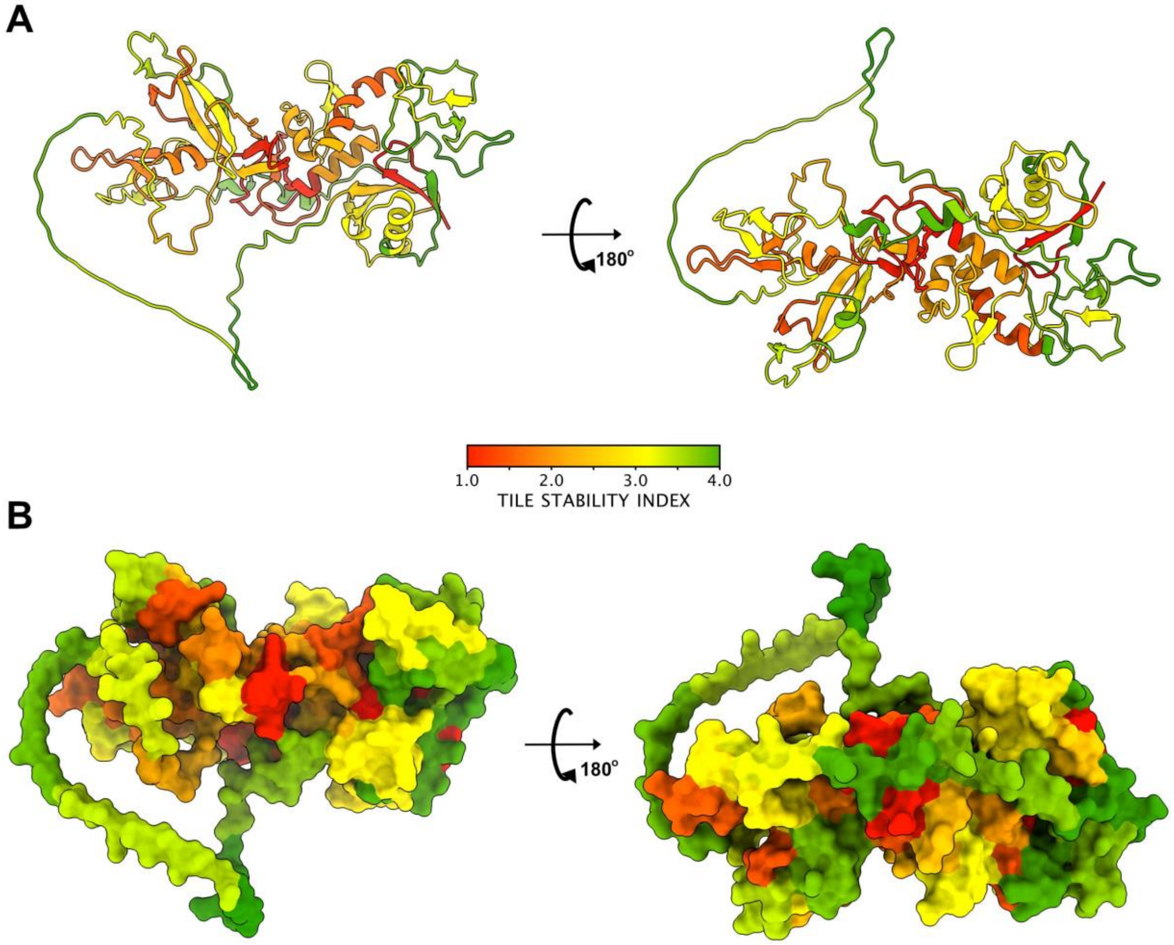

Supplementary Figure 10 – *Low stability tiles are buried in the Parkin structure.*
(A) Cartoon and (B) space filling representations of the Parkin structure colored by TSI. Note that tiles with low stability are mostly buried inside the structure of the full-length protein.

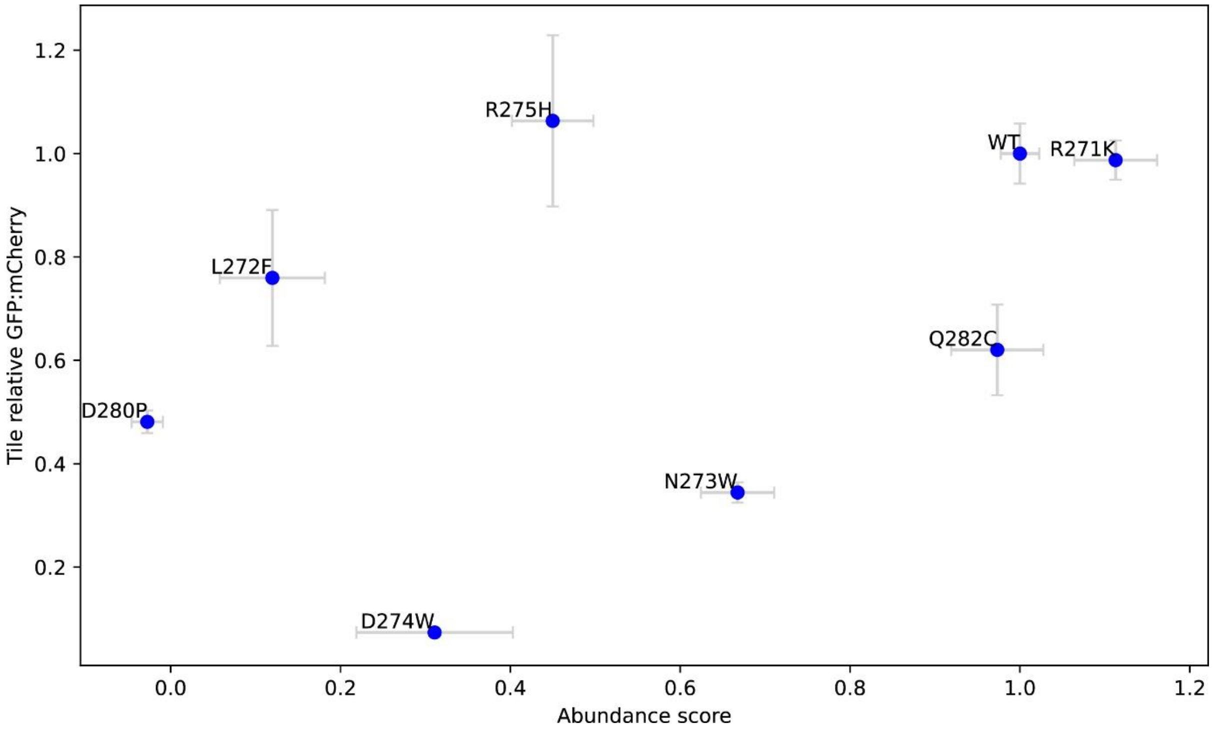

Supplementary Figure 11 – *Effect of mutations in a buried Parkin tile.*

Scatterplot comparing relative GFP:mCherry ratios determined individually for selected tile 23 missense variants in low-throughput by flow cytometry normalized to the WT tile 23 (n=3) and abundance scores derived from the VAMP-seq experiment for full-length Parkin variants. Error bars show the standard deviation. As expected, effects of substitutions in a tile that is buried in the full-length protein correlate weakly (Spearman's r: 0.40) compared to tiles that are exposed in the native protein structure (as presented in Fig. 4F).

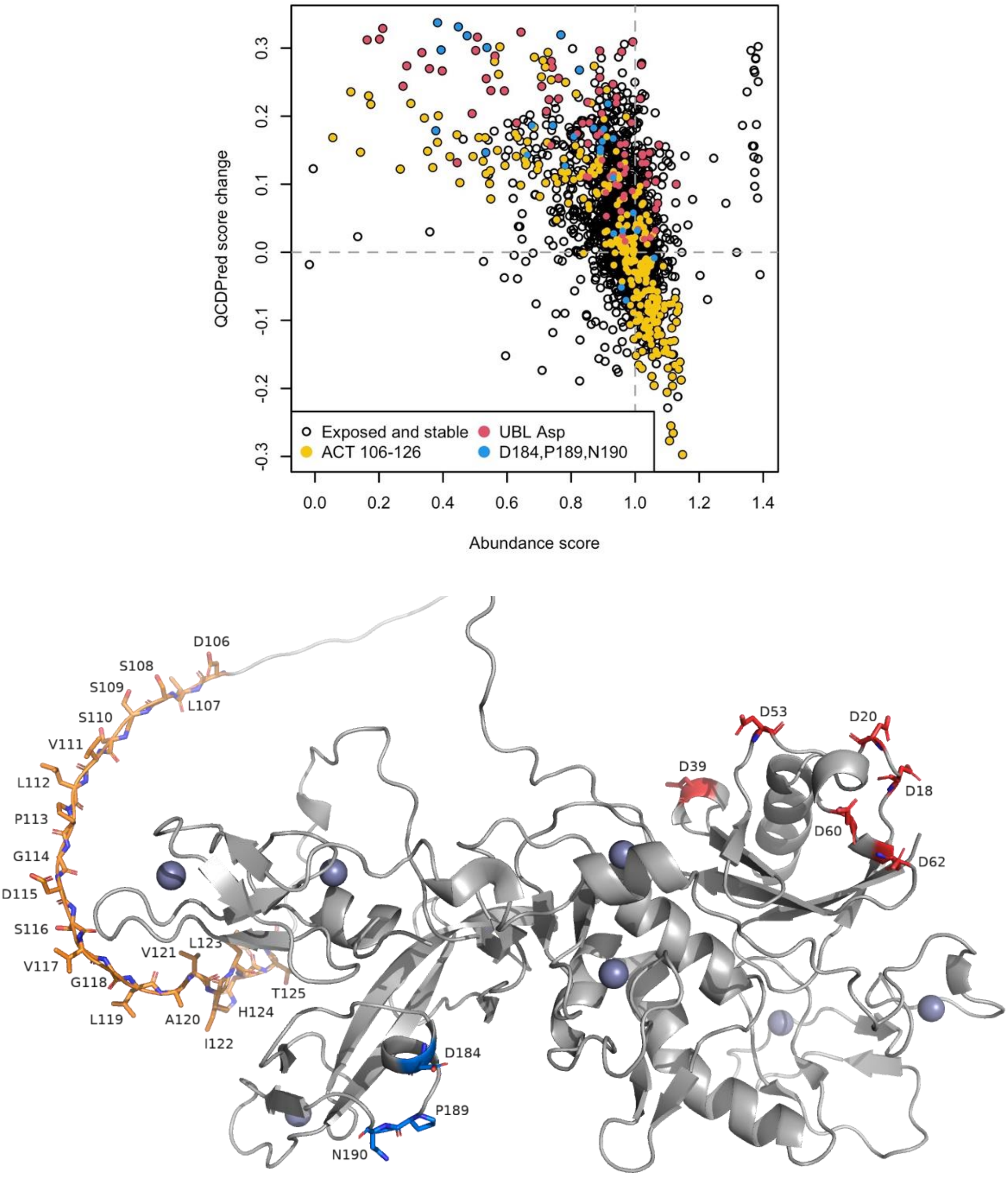

Supplementary Figure 12 – *Predicted degradation signals in low-abundance variants.*

Top: Scatter plot of 2163 solvent exposed (WCN<7.0) and stable (Rosetta ΔΔG<1.0 kcal/mol) variants (Spearman's r: -0.49). Of the 63 variants with low abundance (score < 0.6), 28 also have QCDPred score increased by 0.2 or more (44% of 63 stable and exposed variants or ~1% of all 2722 low-abundance variants in the screen), suggesting that the mechanism for the lower abundance is that these variants become better substrates for the general PQC system. This effect is observed to be strongest in three regions: A patch of the ACT element (yellow), the aspartic acids of the UBL domain (red), and a small patch in the RING0 domain (blue). The effect is strongest for D20, D39, D53, D106, G118 and D184. The wild-type ACT patch already has some PQC activity and variants here with decreased QCDPred score are observed to become more abundant than the wild type. Bottom: Structural positions of the residues mentioned in the top plot shown in the AlphaFold model where the disordered loop is visualized as an extended coil.

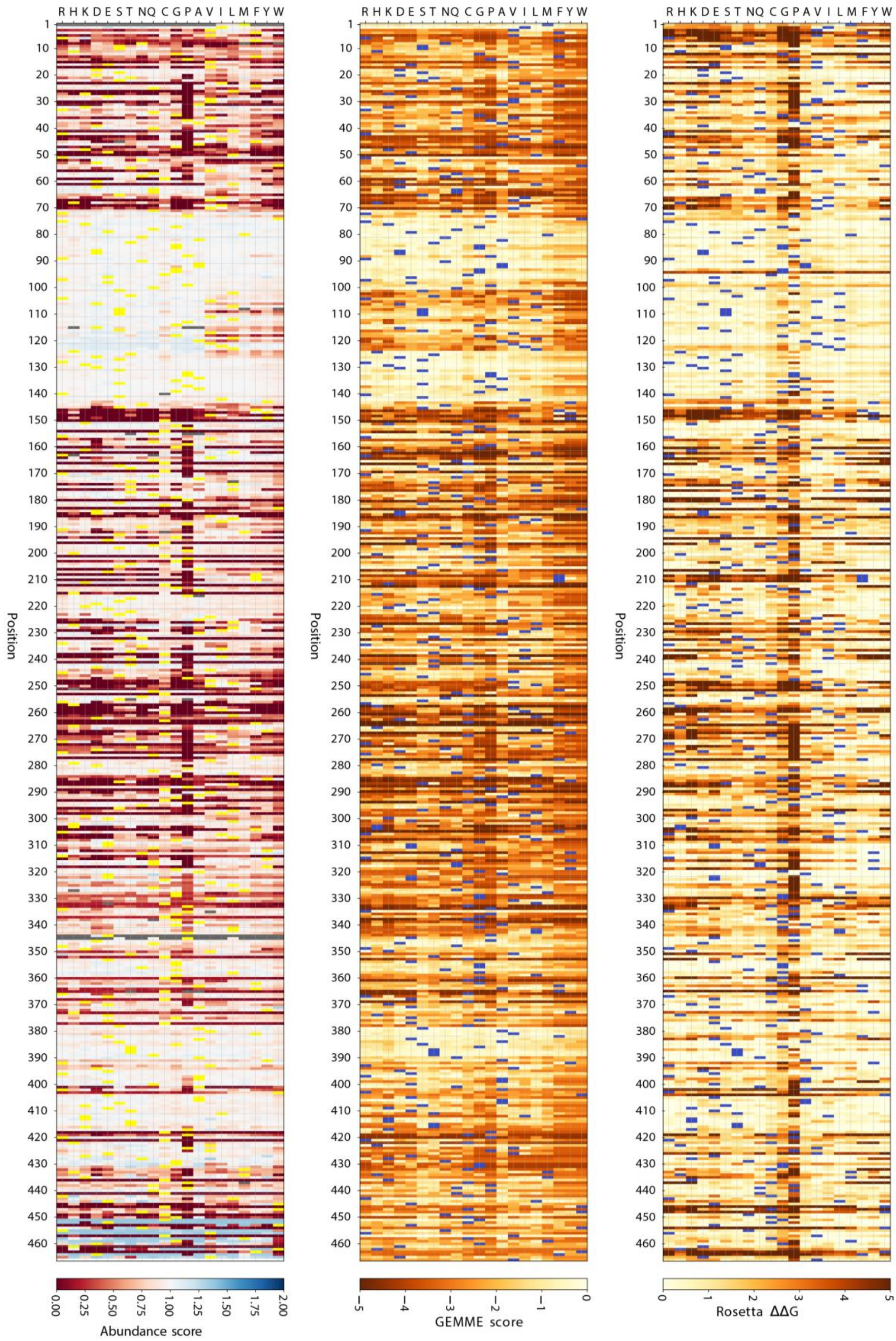

Supplementary Figure 13 - *Side-by-side comparison of the abundance map with the computational maps.*

The figure depicts the abundance map as determined by VAMP seq (left) next to the *in silico* maps based on evolutionary conservation by GEMME (middle) and structural stability (ΔΔG in kcal/mol)

determined by Rosetta (right). The abundance map is colored based on the abundance scores ranging from low abundance (red) over WT-like abundance (white) to increased abundance (blue). Dark grey indicates missing variants. Yellow indicates the wild-type residue. The GEMME and Rosetta maps are colored based on the scores from neutral (white/yellow) to detrimental (orange). The wild-type residues are marked in blue.

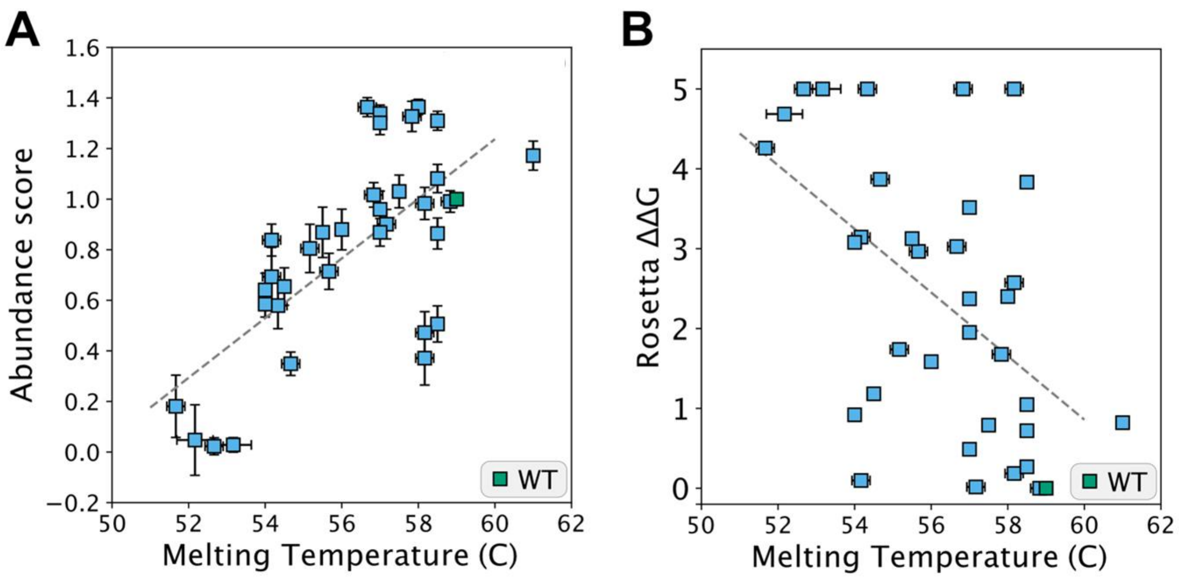

Supplementary Figure 14 – *Correlation with Parkin melting temperatures.*
Correlation of Parkin variant (A) abundance scores and (B) Rosetta ΔΔG (in kcal/mol) with their corresponding melting temperatures determined by Stevens *et al.* [2]. The Spearman's r is: 0.60 in (A) and -0.54 in (B).

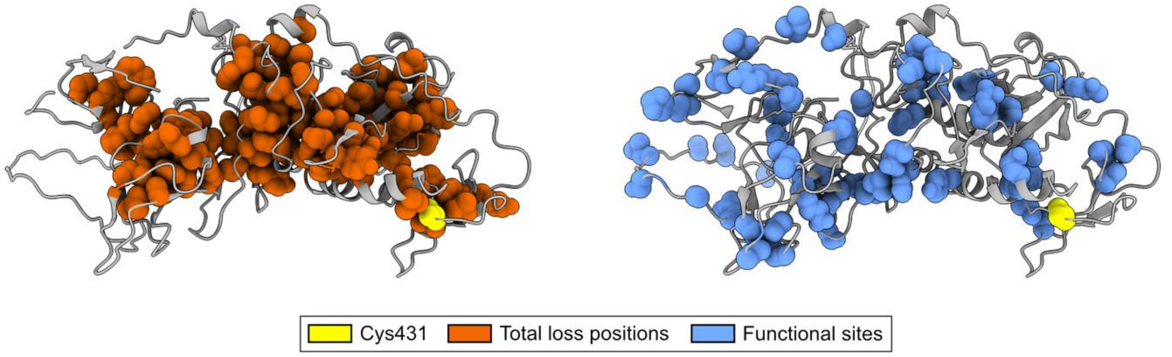

Supplementary Figure 15 – *Functional model classes mapped to the Parkin structure*
Cartoon representation of the Parkin structures with positions classified as total loss shown in red, positions classified as functional sites shown in blue, and the active site cysteine at position 431 (yellow).

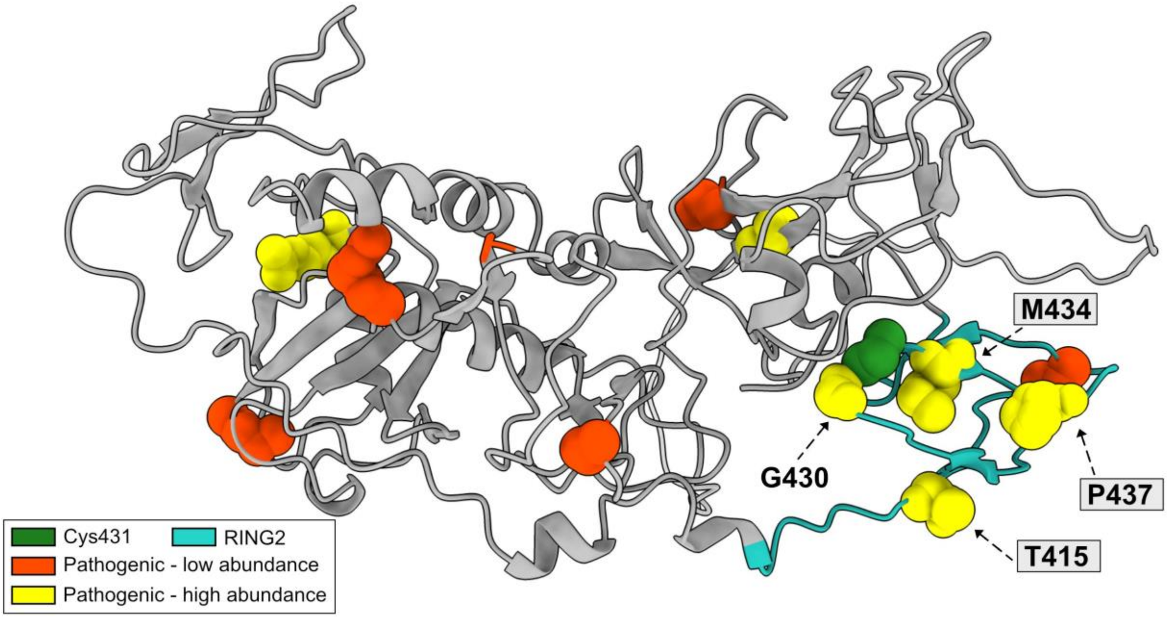

Supplementary Figure 16 – *Positioning of the pathogenic variants in the Parkin structure.*
Cartoon representation of the Parkin structure with pathogenic low abundance (red) and high abundance (yellow) variants marked, along with the active site cysteine at position 431 (green). The catalytic RING2 domain is marked in cyan. Note that several of the pathogenic high abundance variants cluster near the active site in the RING2 domain.

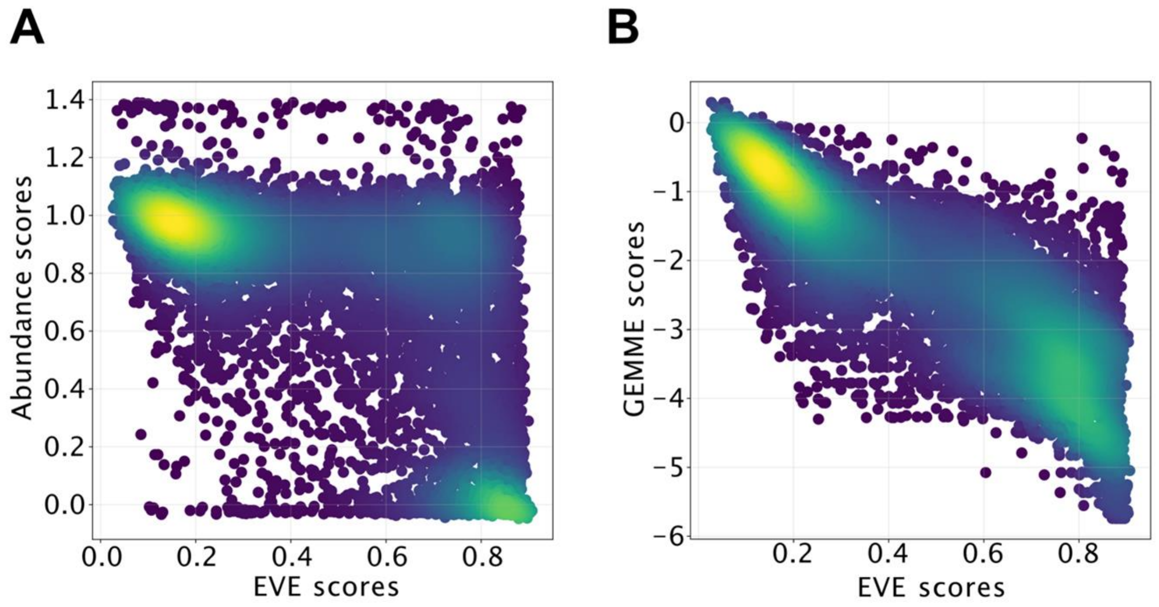

Supplementary Figure 17 – *Correlations with EVE predictions.*

(A) Density scatter plot comparing EVE variant score with experimental abundance scores. The Spearman's r is: -0.61, the confidence interval is: [-0.58,-0.62]. (B) Correlation between the GEMME scores and EVE scores. The Spearman's r is: -0.83, the confidence interval is: [-0.81,-0.83].

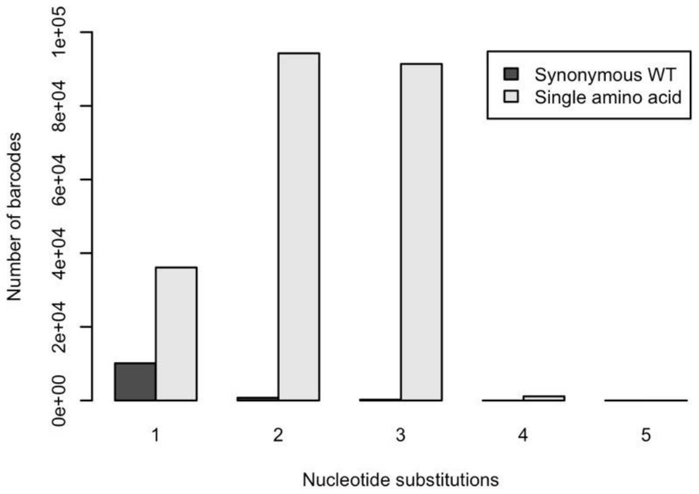

Supplementary Figure 18 – *Distribution of the number of nucleotide substitutions in the library.* The number of nucleotide substitutions in synonymous wild-type variants (black) and single amino acid variants (white) observed in the long-read sequencing. Only 1% of barcodes mapping to single amino acid variants have silent/synonymous mutations, *i.e.*, nucleotide substitutions outside the codon that change.

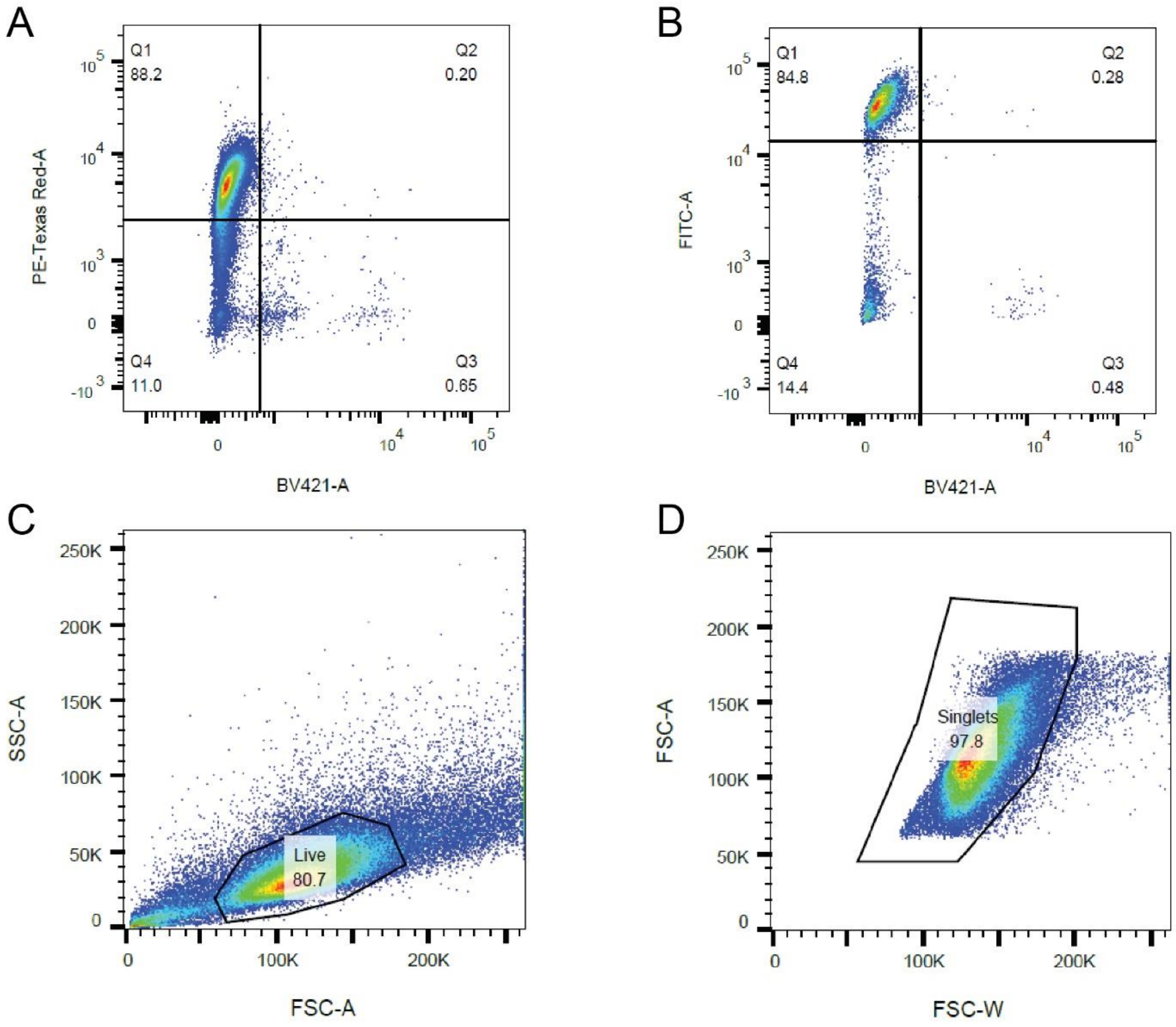

Supplementary Figure 19 – *Flow cytometry gating strategy.*

(A) For abundance measurements and VAMP seq. (all flow cytometry data presented in the manuscript except for those presented in supplemental Fig. 1), the mCherry positive and BFP negative cells were gated, corresponding to the Q1 quadrant. The shown data are for GFP-tagged Parkin library. (B) For the mtKeima-based activity assay (presented in supplemental Fig. 1), the GFP positive and BFP negative cells were gated, corresponding to the Q1 quadrant. The shown data are for wild-type GFP-tagged Parkin. For all flow cytometry experiments, back-gating was applied to select for: (C) live cells (the shown data are for GFP-tagged Parkin library) and (D) singlets (the shown data are for GFP-tagged Parkin library) based on forward and side scattering.

Supplementary Table 1

Data for the pathogenic and benign variants included in this study

Variant position	VAMP score	GEMME score	Rosetta ΔΔG score (kcal/mol)	gnomAD allele freq.	ClinVar category	Notes
Wild-type	1	0	0	-	Benign	-
D18N	0.99	-0.09	0.51	3.40E-04	Benign	-
R33Q	0.84	-0.41	0.23	7.85E-05	Pathogenic	-
Q34R	0.90	-1.66	0.90	3.05E-03	Benign	-
R42C	1.02	-1.46	0.56	1.47E-04	Benign	-
R42P	-0.02	-3.65	3.85	2.53E-05	Pathogenic	-
A46T	0.65	-1.58	2.12	3.17E-03	Benign	-
A82E	1.03	-0.15	-0.50	3.06E-03	Conflicting	Reclassified, benign*
P153R	0.98	-1.98	1.11	2.77E-03	Benign	-
K161N	0.94	-3.98	1.50	6.98E-06	Pathogenic	-
S167N	0.95	-0.33	0.66	0.06	Benign	-
M192L	1.02	-0.93	-0.09	0.01	Benign	-
C212Y	-0.01	-5.27	6.41	1.19E-05	Pathogenic	-
V248I	0.92	-1.40	0.55	1.17E-04	Benign	-
C253Y	-0.03	-4.55	2.61	1.59E-05	Pathogenic	-
L272I	0.91	-1.77	0.78	7.60E-05	Benign	-
R275W	-0.02	-3.57	1.73	2.02E-03	Pathogenic	-
G284R	0.44	-4.85	2.00	1.19E-05	Pathogenic	-
R334C	0.48	-0.87	0.75	1.72E-03	Conflicting	Reclassified, benign*
R366W	0.74	-4.03	0.57	2.23E-04	Benign	-
V380L	0.92	-0.06	0.44	0.17	Benign	-
D394N	0.85	-0.25	0.14	0.03	Benign	-
R402C	0.96	-1.54	1.38	1.91E-03	Conflicting	Reclassified, benign*
T415N	1.02	-3.19	0.73	7.95E-06	Pathogenic	-
G430D	1.00	-3.59	-1.07	8.42E-05	Pathogenic	-
M434T	1.17	-2.81	2.87	#	Pathogenic	-
P437L	0.87	-1.12	0.77	1.80E-03	Conflicting	Reclassified, pathogenic**
C441R	-0.02	-3.40	1.61	5.20E-05	Pathogenic	-

#= variant not listed in gnomAD.

*= assigned new category based on Sherloc criteria [1,3].

**= assigned new category based on MDSgene database [4].

Supplementary Table 2

Primers used in this study

Name	Sequence
ASPA_PARK2_index2_re	ACGCAATTGCAGAACTAGTCCTCATATGTCCTGG
gDNA_2nd	AATGATACGGCGACCACCGAGATCTACAC(NNNNNNNN)CGTGACCGCCGCC
JS_R	CAAGCAGAAGACGGCATACGAGAT(NNNNNNNN)GGGTTAGCAAGTGGCAGCCT
LC1020	CCAGGACATATGAGGACTAG
LC1031	GGGTTAGCAAGTGGCAGCCTTCTCCTTAATCAGCTCTTCG
LC1040	AAGAACCGCTAGAAGCGTCGCTGTACAAATAGTT
LC1041	CGAGAAAGCTAGCGCAAACGACTACTCGCA
LC1042	CTGATTAAGGAGAAGGCTGCCACTTGCTAACCC
PCR2_Fw	AATGATACGGCGACCACCGAGATCTACAC(NNNNNNNN)CCAGGACATATGAGGACTAG
VV1	TAGTAACTTAAGAATTCACCGGTCTGACCT
VV2	GGTGGCTCCGCTGCCTCTA
VV2S	GGGTTAGCAAGTGGCAGCCTTGCAGACCTGAAGAGAGGGA
VV3	AAGACGCGTTCTAGAGGCAGC
VV4	AGGTCAGACCGGTGAATTCTTAAGTTACTA
VV16	CGGTCACGAACTCCAGCAGGACCATGTG
VV18	GGGAGAAGGAGGTCAGACCGGTGAATTCTTAAGTTACTA
VV19	TCTGCAAGGCTGCCACTTGCTAACCC
VV21	GAGTGATCCCGGCGGCGGTCACG
VV40S	GAGAACGTATGTCGAGGTAGGC

References for supplementary information

1. Yi, W. *et al.* The landscape of Parkin variants reveals pathogenic mechanisms and therapeutic targets in Parkinson's disease. *Hum Mol Genet* **28**, 2811 (2019).

2. Stevens, M. U. *et al.* Structure-based design and characterization of Parkin-activating mutations. *Life Sci Alliance* **6**, e202201419 (2023).

3. Nykamp, K. *et al.* Sherloc: a comprehensive refinement of the ACMG-AMP variant classification criteria. *Genet Med* **19**, 1105–1117 (2017).

4. Lill, C. M. *et al.* Launching the movement disorders society genetic mutation database (MDSGene). *Movement Disorders* **31**, 607–609 (2016).
