# Supplementary material for: A mutational atlas for Parkin proteostasis
Source: Nat Commun. 2024 Feb 20;15:1541. doi: 10.1038/s41467-024-45829-4 (PMC10879094; doi:10.1038/s41467-024-45829-4)
Supplement: Supplementary file 3 — Reporting Summary [file 41467_2024_45829_MOESM3_ESM.pdf]

Corresponding author(s): Rasmus Hartmann-Petersen
Kresten Lindorff-Larsen
Douglas M. Fowler

Last updated by author(s): Dec 11, 2023

Reporting Summary

Nature Portfolio wishes to improve the reproducibility of the work that we publish. This form provides structure for consistency and transparency in reporting. For further information on Nature Portfolio policies, see our Editorial Policies and the Editorial Policy Checklist.

Statistics

For all statistical analyses, confirm that the following items are present in the figure legend, table legend, main text, or Methods section.

n/a	Confirmed	
☐	☒	The exact sample size (n) for each experimental group/condition, given as a discrete number and unit of measurement
☐	☒	A statement on whether measurements were taken from distinct samples or whether the same sample was measured repeatedly
☒	☐	The statistical test(s) used AND whether they are one- or two-sided<br>*Only common tests should be described solely by name; describe more complex techniques in the Methods section.*
☒	☐	A description of all covariates tested
☒	☐	A description of any assumptions or corrections, such as tests of normality and adjustment for multiple comparisons
☐	☒	A full description of the statistical parameters including central tendency (e.g. means) or other basic estimates (e.g. regression coefficient) AND variation (e.g. standard deviation) or associated estimates of uncertainty (e.g. confidence intervals)
☒	☐	For null hypothesis testing, the test statistic (e.g. F, t, r) with confidence intervals, effect sizes, degrees of freedom and P value noted<br>*Give P values as exact values whenever suitable.*
☒	☐	For Bayesian analysis, information on the choice of priors and Markov chain Monte Carlo settings
☒	☐	For hierarchical and complex designs, identification of the appropriate level for tests and full reporting of outcomes
☐	☒	Estimates of effect sizes (e.g. Cohen's d, Pearson's r), indicating how they were calculated

Our web collection on statistics for biologists contains articles on many of the points above.

Software and code

Policy information about availability of computer code

Data collection	Pacbio reads from the two sequencing runs were merged and aligned to the barcode-GFP-PRKN construct using BWA and the barcode and PRKN sequences were extracted using cutadapt, see pacbio/pacbio_align.sh available at GitHub. Reads comprising ten or more DNA substitutions or any indels were removed and in cases where the same barcode mapped to more PRKN variants, the variant having most read counts was used to make a unique map of each barcode.<br>Illumina sequencing reads were converted to fastq format and de-multiplexed with Basespace (Illumina).
Data analysis	Illumina reads were cleaned for adapters sequences using cutadapt and paired end reads were joined using fastq-join from ea-utils, see illumina/call_zerotol_paired.sh available at GitHub. Only barcodes with a perfect match to the barcode map were counted, see illumina/merge_counts.r. Barcode counts for the 48 pairs of technical replicates have an average Pearson correlation of 0.82 (range 0.71-0.94) for the 257,610 unique barcodes and an average of 2.1 mill. matched reads (range 1.3-4.8 mill. reads) per technical replicate. Technical replicates of each FACS bin were merged and normalized to frequencies without pseudo counts. The frequencies were used to calculate a protein stability index (PSI). Barcode PSIs were averaged per amino acid variant and finally, the PSIs of all replicates were averaged and normalized to generate an abundance. The standard deviation per variant is reported as an error estimate in the data file (SupplementalFile1.xlsx).<br>TSIs were generated as above. Thus, the tile reads were cleaned for adapters sequences using cutadapt and paired end reads were joined using fastq-join from ea-utils, see illumine_degron/call_zerotol_paired.sh available at GitHub. Only barcodes having a perfect match to the barcode map were counted, see illumine_degron/merge_counts.r. When tiles from the Odds, Evens or CT libraries were noticed in a sorting of a different library, these were considered to be non-sorted contaminants and disregarded. Technical replicates of each FACS bin were merged and normalized to frequencies having no pseudo counts. Each tile was on average covered by more than 3500 detected reads per technical replicate. Thus, 3 biological and 2 technical/FACS replicates for each of the 3 libraries generated TSI scores with a minimum Pearson correlation of 0.97. The standard deviation per tile is noted as an error estimate in the data file (SupplementalFile1.xlsx).<br>All data and software generated for this article is available on GitHub: https://github.com/KULL-Centre/_2023_Clausen_parkin_MAVE

(DOI:10.5281/zenodo.8009574)
Additional code that we used include:
Rosetta (GitHub SHA1 99d33ec59ce9fcecc5e4f3800c778a54afdf8504)
GEMME v1.0

For manuscripts utilizing custom algorithms or software that are central to the research but not yet described in published literature, software must be made available to editors and reviewers. We strongly encourage code deposition in a community repository (e.g. GitHub). See the Nature Portfolio guidelines for submitting code & software for further information.

Data

Policy information about availability of data

All manuscripts must include a data availability statement. This statement should provide the following information, where applicable:

- Accession codes, unique identifiers, or web links for publicly available datasets
- A description of any restrictions on data availability
- For clinical datasets or third party data, please ensure that the statement adheres to our policy

All data and software generated for this article is available on GitHub: https://github.com/KULL-Centre/_2023_Clausen_parkin_MAVE (DOI:10.5281/zenodo.8009574). Abundance scores are also deposited at MaveDB.org (entry urn:mavedb:00000114). Sequencing reads for the abundance scores are available at https://doi.org/10.17894/ucph.ef2e30c5-d262-4713-86e8-a3964b5dd6c7 and for the degron scores https://doi.org/10.17894/ucph.d879cfce-efb3-4eaa-928f-87a94d9560ef.
In addition, data was used from MDSGene (https://www.mdsgene.org/), ClinVar (https://www.ncbi.nlm.nih.gov/clinvar/), and EVE (https://evemodel.org/).

Research involving human participants, their data, or biological material

Policy information about studies with human participants or human data. See also policy information about sex, gender (identity/presentation), and sexual orientation and race, ethnicity and racism.

Reporting on sex and gender	Not applicable
Reporting on race, ethnicity, or other socially relevant groupings	Not applicable
Population characteristics	Not applicable
Recruitment	Not applicable
Ethics oversight	Not applicable

Note that full information on the approval of the study protocol must also be provided in the manuscript.

Field-specific reporting

Please select the one below that is the best fit for your research. If you are not sure, read the appropriate sections before making your selection.

☒ Life sciences ☐ Behavioural & social sciences ☐ Ecological, evolutionary & environmental sciences

For a reference copy of the document with all sections, see nature.com/documents/nr-reporting-summary-flat.pdf

Life sciences study design

All studies must disclose on these points even when the disclosure is negative.

Sample size	No sample size calculations were performed. To ensure reproducibility and full coverage of the library the following measures were taken: The VAMP seq. screening was performed 12 times in total: 4 biological repeats (separate library transfections/selections B1-4), with 3 repeats of the cell sorting for each (FACS1-3). At all steps we made sure to maintain 100 fold coverage of the library complexity (i.e. at least 9300 variants x 100 = 930,000). For the tile sequencing we performed 3 biological replicates (separate library transfections/selections), with 2 repeats of the cell sorting for each. We aimed for a 1000 fold coverage of the library complexity (i.e. at least 194 x 1000 = 194,000).<br>The correlations between the repeats and >99% obtained coverage, indicate that the above measures were sufficient. This is in agreement with previous VAMP-seq screens (Matreyek et al. Nat. Genet. 50:874-882 (2018)).
Data exclusions	As described in the methods section, for PacBio reads comprising ten or more DNA substitutions or any indels were removed and in cases where the same barcode mapped to more PRKN variants. Only barcodes with a perfect match to the barcode map were counted, as these are the only ones where we can be sure in the variant identification. These criteria were pre-established.
Replication	All attempts at replication were successful and are included in the paper.<br>The VAMP seq. screening was performed 12 times in total: 4 biological repeats (separate library transfections/selections B1-4), with 3 repeats of the cell sorting for each (FACS1-3).

For the tile sequencing we performed 3 biological replicates (separate library transfections/selections), with 2 repeats of the cell sorting for each.

Randomization	No samples/organisms/participants were allocated into groups. This was not possible with the experimental design employed.
Blinding	There was no group allocations. Blinding was unnecessary as the identity of the variants was unknown during the experiment.

Reporting for specific materials, systems and methods

We require information from authors about some types of materials, experimental systems and methods used in many studies. Here, indicate whether each material, system or method listed is relevant to your study. If you are not sure if a list item applies to your research, read the appropriate section before selecting a response.

Materials & experimental systems

n/a	Involved in the study	
☐	☒	Antibodies
☐	☒	Eukaryotic cell lines
☒	☐	Palaeontology and archaeology
☒	☐	Animals and other organisms
☒	☐	Clinical data
☒	☐	Dual use research of concern
☒	☐	Plants

Methods

n/a	Involved in the study	
☒	☐	ChIP-seq
☐	☒	Flow cytometry
☒	☐	MRI-based neuroimaging

Antibodies

Antibodies used	Rat anti-GFP, Western blotting, Chromotek, 3H9, 3H9, diluted 1:1000;<br>Mouse anti-mCherry/RFP, Western blotting, Chromotek, 6G6, 6G6, diluted 1:1000;<br>Mouse anti-Parkin, Western blotting, Cruz Biotechnology, SC-32282, PRK8, diluted 1:1000;<br>Rabbit anti-GAPDH, Werstern blotting; Cell Signaling Technology, 14C10, diluted 1:1000
Validation	The anti-Parkin, anti-GFP, anti-mCherry antibodies are validated (show no reaction in untransfected control lysates) in the manuscript. The other antibody was used as a loading control and showed the expected reactivity.<br>Additional validation data for the antibodies are available from the following company websites:<br>https://www.ptglab.com/products/GFP-antibody-3H9.htm<br>https://www.ptglab.com/products/RFP-antibody-6G6.htm<br>https://www.scbt.com/p/parkin-antibody-prk8<br>https://www.cellsignal.com/products/primary-antibodies/gapdh-14c10-rabbit-mab/2118?_requestid=1720734

Eukaryotic cell lines

Policy information about cell lines and Sex and Gender in Research

Cell line source(s)	The HEK293T cells containing the landing pad are from: Matreyek et al. (2020) Nucleic Acids Res. 48:e1. doi: 10.1093/nar/gkz910.
Authentication	Authentication was regularly confirmed by selection with AP1903 and checking for expression of BFP from the Tet-on promoter in non-recombinant cells. No other authentications were performed.
Mycoplasma contamination	The cells tested negative for mycoplasma.
Commonly misidentified lines<br>(See ICLAC register)	No commonly misidentified cells were used.

Flow Cytometry

Plots

Confirm that:

☒ The axis labels state the marker and fluorochrome used (e.g. CD4-FITC).

☒ The axis scales are clearly visible. Include numbers along axes only for bottom left plot of group (a 'group' is an analysis of identical markers).

☒ All plots are contour plots with outliers or pseudocolor plots.

☒ A numerical value for number of cells or percentage (with statistics) is provided.

Methodology

Sample preparation	Dislodged HEK293 cells in (5% v/v) bovine calf serum in PBS filtered through a 35 micrometer nylon mesh.
Instrument	Cells were sorted on a BD Biosciences ARIA III instrument equipped with a 70 micrometer nozzle. The laser used for excitation of BFP was 405 nm, for GFP 488 nm and for mCherry 562 nm. The filters used were 442/46 for BFP, 530/30 for GFP and 615/20 for mCherry. For analytical flow cytometry, we used a BD FACSJazz instrument. BFP was excited at 405 nm, GFP and mCherry were excited by a 488 nm laser and a 561 nm laser, respectively. Live single cells were gated using forward and side scatter before successfully recombined cells were gated on BFP negativity and mCherry positivity. The filters were 450/50 for BFP, 530/40 for GFP and 610/20 for mCherry.
Software	FACSDiva<br>FlowJo v10.9.0
Cell population abundance	The cells were sorted into 4 equally populated bins based on the GFP/mCherry ratios. All the sorted cells were used for DNA sequencing. The controls (WT and R42P) were located in the expected bins. The VAMP seq. data correlated with low throughput validation of individual variants.
Gating strategy	1) The population of cells was gated by using FSC-A and SSC-A (for live cells) and FSC-A and FSC-H (for single cells).<br>2) The recombinant cells were selected based on their lack of BFP, and expression of mCherry. The mCherry signal was at least 10 times higher than the median fluorescence value of negative or control cells, and the BFP fluorescence signal was at least 10 times lower than the median of the unrecombined BFP positive cells.<br>3) A histogram of the GFP:mCherry ratiometric parameter was established on the FACSDiva software and gates were set to separate the whole library into four equally populated bins based on the GFP:mCherry ratio. An example of the gating strategy is shown in Fig. 1F.

☒ Tick this box to confirm that a figure exemplifying the gating strategy is provided in the Supplementary Information.
